# Supplementary material for: Tuning the Coherent Propagation of Organic Exciton‐Polaritons through Dark State Delocalization
Source: Adv Sci (Weinh). 2022 Apr 27;9(18):2105569. doi: 10.1002/advs.202105569 (PMC9218652; doi:10.1002/advs.202105569)
Supplement: Supplementary file 1 — Supporting Information [file ADVS-9-2105569-s001.pdf]

Tuning the coherent propagation of organic exciton-polaritons through dark state delocalization – SUPPORTING INFORMATION

[1,2]Raj Pandya, [1]Arjun Ashoka, [3,4] Kyriacos Georgiou, [1]Jooyoung Sung, [3]Rahul Jayaprakash, [5]Scott Renken, [6,7]Lizhi Gai, [7]Zhen Shen, [1]Akshay Rao and [5]Andrew Musser*

[1]Cavendish Laboratory, University of Cambridge, J.J. Thomson Avenue, CB3 0HE, Cambridge, United Kingdom

[2]Laboratoire Kastler Brossel, École Normale Superiéure-Université PSL, CNRS, Sorbonne Université, College de France, Paris 75005, France

[3] Department of Physics and Astronomy, University of Sheffield, Sheffield S3 7RH, United Kingdom

[4] Department of Physics, University of Cyprus, P.O. Box 20537, Nicosia, 1678, Cyprus

[5] Department of Chemistry and Chemical Biology, Cornell University, 14853 Ithaca, NY, USA

[6] Key Laboratory of Organosilicon Chemistry and Material Technology, Ministry of Education, Hangzhou Normal University, Hangzhou, 311121, China

[7] State Key Laboratory of Coordination and Chemistry, School of Chemistry and Chemical Engineering, Nanjing University, Nanjing, 210046, China

correspondence: ajm557@cornell.edu

Table of Contents

S1: Optical modelling of microcavities 2

S2: LP photoinduced absorption dynamics 5

S3: Fluence dependence of BODIPY-R and microcavity dynamics 6

S4: Extraction of $\boldsymbol{\sigma(t)}$ 7

S5: Pump-probe spot size at t_0 8

S6: Modelling coherent polariton propagation 11

References 15

S1: Optical modelling of microcavities

We describe the optical properties of our microcavities using a transfer matrix model, the basis of which is described in detail in the "Basics of optics of multilayer systems".[1] We treat the microcavity as a series of isotropic, homogeneous layers, each defined by a unique complex refractive index n and thickness d. As input to the model, the absorption spectrum of the organic active layer was fitted using a series of Lorentzian functions, with the amplitude of the functions describing the excitonic oscillator strength. From fitting to the absorption spectrum, we determine the complex refractive index of the exciton absorption and superimpose this on the background refractive index of the polystyrene host. The incident beam is assumed to be propagating from the air towards the substrate at an angle of incidence θ, suffering multiple reflections/transmissions at each interface, interfering either constructively or destructively at the output, which can be either at the reflected side or the transmitted side. The model separately determines the characteristic matrix of each layer based on its material properties, and due to the continuity of the tangential components of the electric and magnetic fields at each interface we can determine the effective characteristic matrix of the total structure as the product of the single-layer characteristic matrices. This model was used to simulate the angle-dependent reflectivity and PL emission from the planar microcavity structure.

In order to minimize optical artefacts and oxygen-related degradation processes, the samples for TA microscopy were prepared on thin microscope coverslips and immediately encapsulated in inert atmosphere. This prevented them from fitting into our reflectivity goniometer to directly characterize the dispersion, so instead we describe their optical properties based on the measured properties of organic thin films made from the same solution batch and individual DBRs taken from the same deposition runs. The spectra of these components are illustrated in main-text Figure 1, and they can be well described by our model. We can thus fully model the reflectivity dispersions of our cavity Q-factor series, as illustrated in Figure S1 for an active layer thickness of 215 nm. The positions of the modulations detected in transient absorption, as reported in main-text Figure 1e, confirm the general picture suggested by this model. We note that penetration into the DBRs contributes to the cavity length in such structures; the invariance of the polariton dispersions through this series indicates that this penetration effect is the same for n=3.5 through n=6.5. To define the Q-factor of our structures, we turn to an equivalent optical model for each cavity with the dye absorption strength set to zero. The Q-factor is extracted from these 'empty' cavity models as $Q = \lambda/\Delta\lambda$ at the mode minimum. The corresponding photonic lifetime quoted in the main text is determined through $\tau = \frac{Q\lambda}{2\pi c}$, where λ is the bare photonic mode wavelength at normal incidence.[2]

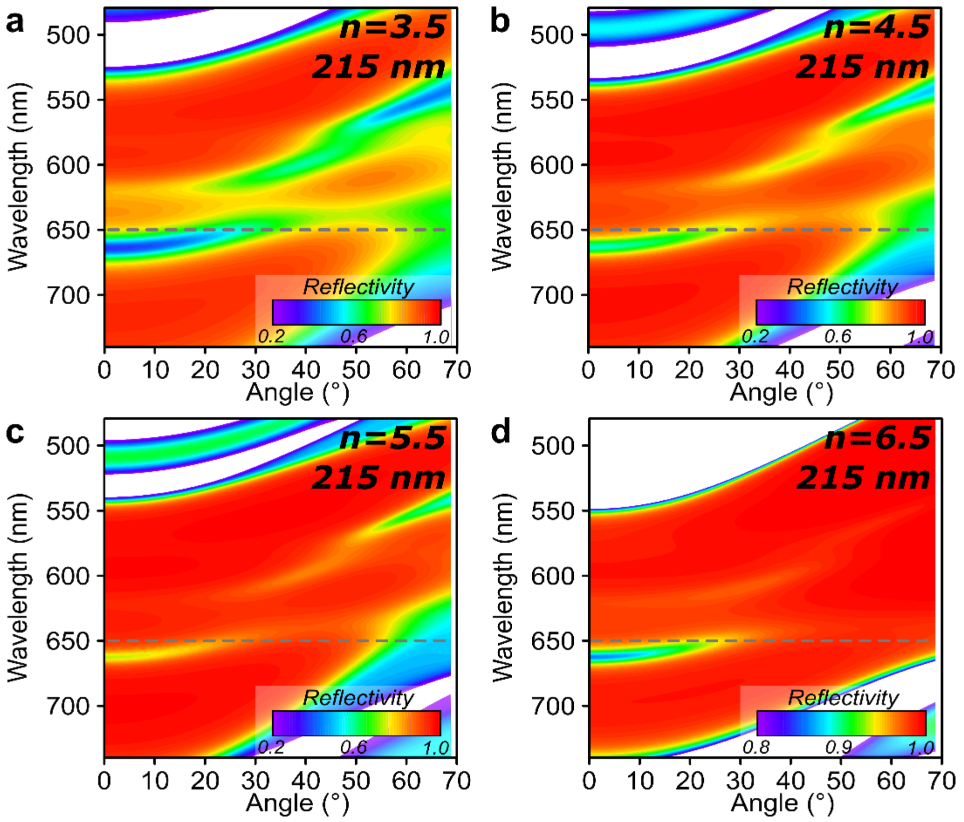

Figure S1. Reflectivity dispersion as a function of cavity Q-factor. Transfer-matrix modelled reflectivity, benchmarked by the experimental data in main-text Figure 1, for microcavities with a BODIPY-R/polystyrene active-layer thickness of 215 nm. Increasing the number of Nb_2O_5/SiO_2 pairs in the top and bottom mirrors, as indicated in each panel, does not affect the dispersion but only the linewidth and relative depth of polaritonic features. Note the change in colour scale in panel d due to the very high reflectivity of those structures. Dashed line at 650 nm is a guide to the eye to highlight the absence of shifts through the series.

Aside from the expected changes in linewidth and total reflectivity, there are no systematic changes as a function of *Q*-factor. The Rabi splitting, detuning, and curvature of the UP and LP branches are fully invariant, suggesting there should be no alteration to transport properties of polaritons along our microcavity series.

Because the solution-processed thin films used as an active layer in our structures inevitably exhibit thickness variation,[3,4] we use the transfer matrix model to establish what range of cavity thicknesses would be consistent with the chief experimental constraint of our TAM measurements, namely the position of the derivative-like feature at 640 nm. The sensitivity of the polariton dispersions to the cavity thickness means this condition strongly constrains the cavity thickness. Our model results indicate this behaviour is consistent with an active-layer thickness of 200-215 nm; as shown in Figure S2, this range entails a roughly 10-nm shift in the LP minimum.

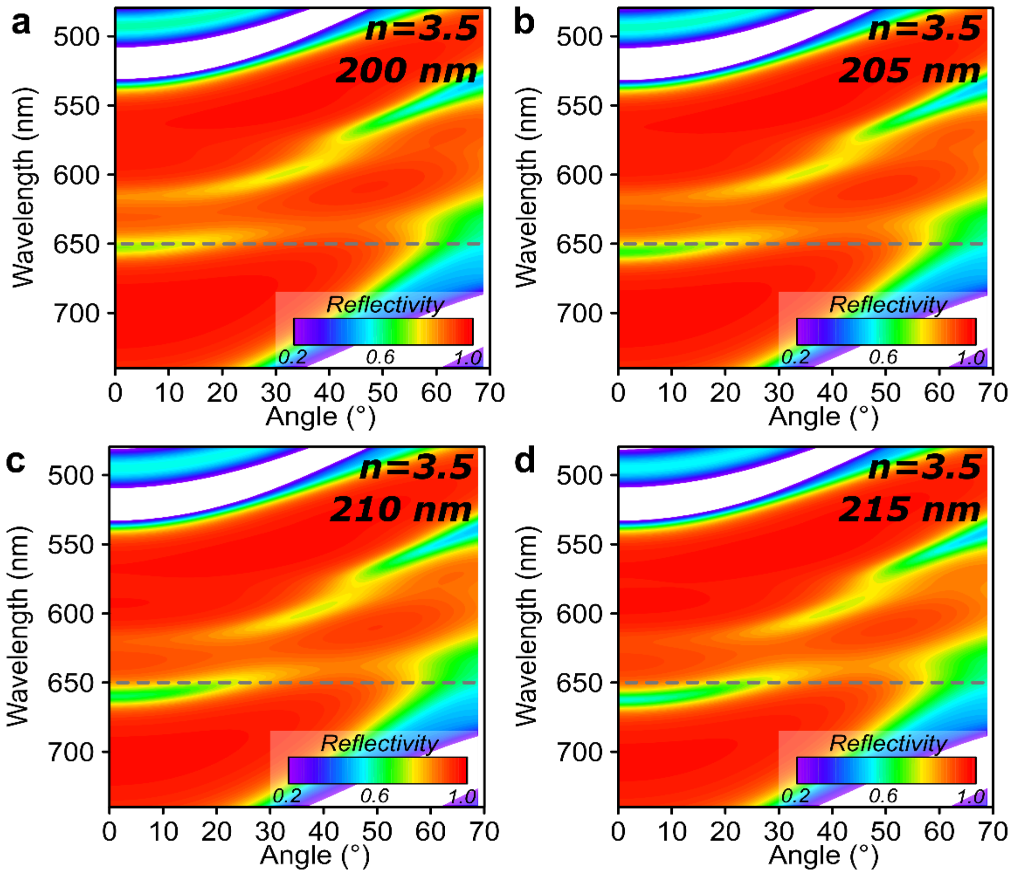

Figure S2. Reflectivity dispersion as a function of cavity thickness. Transfer-matrix modelled reflectivity, benchmarked by the experimental data in main-text Figure 1, for microcavities with 3.5 Nb_2O_5/SiO_2 pairs in the top and bottom mirrors. Active-layer thickness increases from **a** to **d** as indicated in the panels. Increasing the thickness by 15 nm shifts the minimum of the LP branch by >10 nm, easily within the range that can be detected in our instruments, and making or spectral selection in TA microscopy measurements a strong control for cavity thickness. Dashed line at 650 nm is a guide to the eye to highlight the shifts through the series. Similar results are obtained for all other Q-factors considered.

We use the thickness range in Figure S2 as the basis to determine the expected group velocities in our samples. We first use a simple two-state coupled oscillator model to describe the dispersions predicted in our transfer matrix approach. We determine the probability of our wide-angle, broadband excitation source to photoexcite a particular polariton state based on the product of the angle-dependent photonic Hopfield coefficient squared and the intensity of the pump pulse at the corresponding wavelength. The range of *k*-vectors considered spanned from 0 to a *k* value corresponding to the intersection of a free photon at 64 degrees (the limit of our high-NA objective) with the UP or LP dispersion. The integration was also limited by the wavelength range spanned by our excitation pulse (550-630 nm), which reduced the degree of excitation of the lower portion of the LP dispersion. This approach yields polariton population distributions along the LP and UP; these are typically heavily weighted towards the bottom of the LP and the top the UP, where the states are most photonic. At each *k*-vector we determine the expected group velocity and use these population distributions to produce a weighted average velocity for UP versus LP excitation; we keep these separate because the precise contribution of the (much more rapidly moving) UP on the short timescales of our measurement is not well determined. The process was run for structures with 3.5, 4.5, 5.5, and 6.5 mirror pairs. The same procedure was repeated for cavity active layer thicknesses 200-215 nm, which were averaged together at each *Q*-factor to produce the mean and standard deviation results in main-text Figure 2e. Within the limits of the transfer matrix and coupled oscillator models, there is no reason to anticipate any impact of *Q*-factor on polariton group velocity.

S2: LP photoinduced absorption dynamics

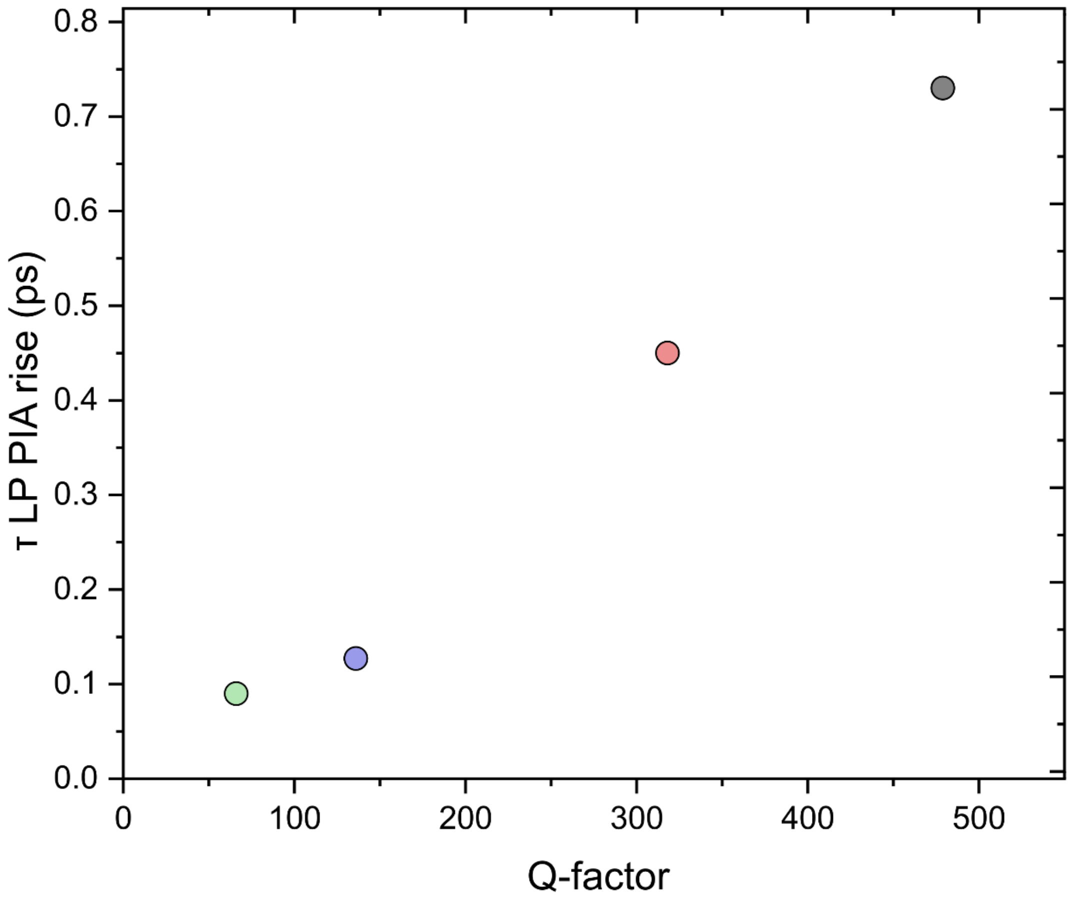

Figure S3. Rise time of LP PIA band as a function of Q-factor. The PIA band slightly redshifted from the steady-state LP position exhibits increasing rise time with increasing Q-factor, in a similar manner to the bleach peaks discussed in Figure 1 of the main text. This strong Q-factor dependence confirms assignment to a polaritonic species.

S3: Fluence dependence of BODIPY-R and microcavity dynamics

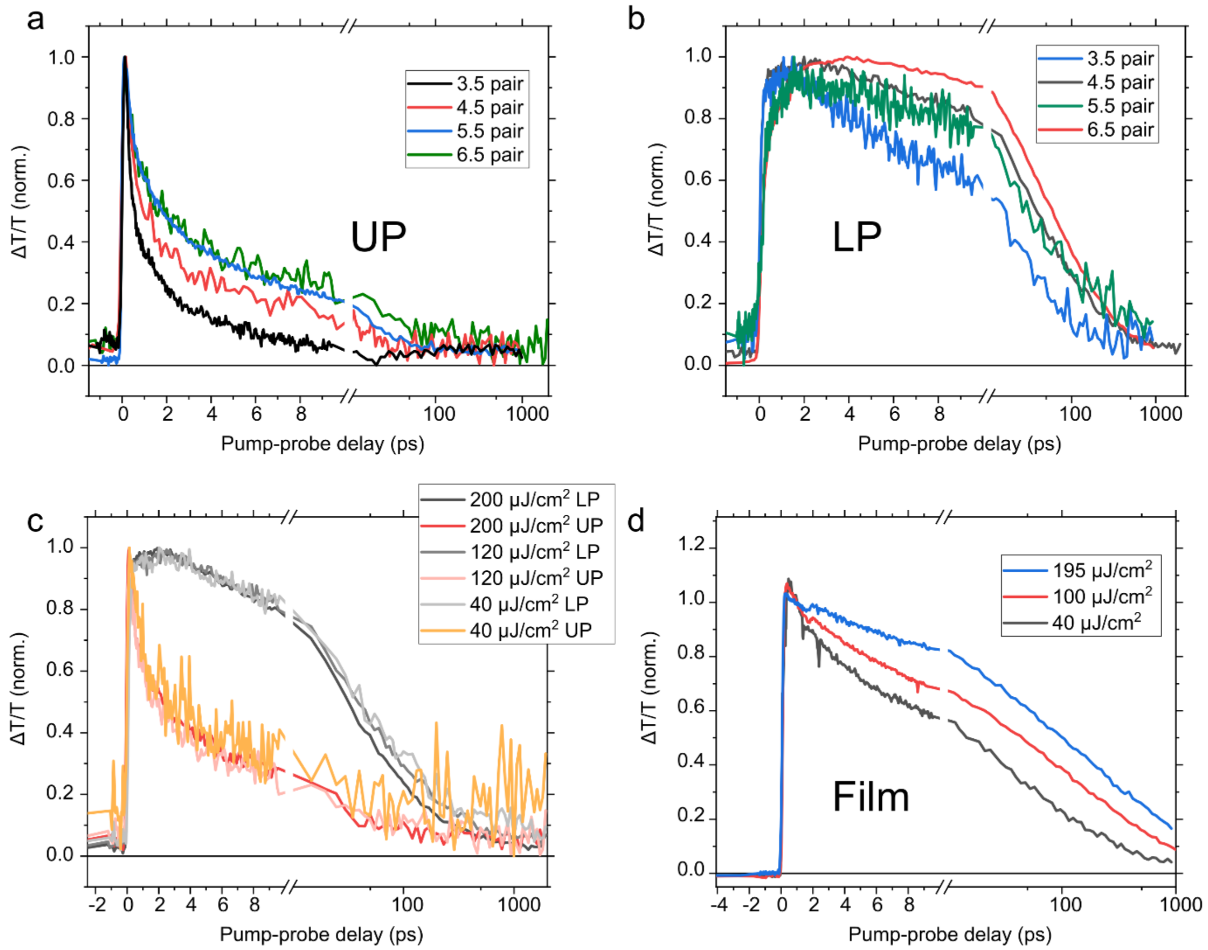

Figure S4. Decay dynamics of R-BODIPY films embedded in microcavities. a-b. In general, the decay time of the signals observed at the spectral position of the upper polariton branch (UP, based on steady-state measurements) increases with an increasing number of SiO_2-Nb_2O_5 mirror pairs (n). This is further reflected in the increased rise and decay times for the signals at the lower polariton branch spectral position (LP, again referred to the steady-state assignment) with an increasing n. We note that on longer timescales (10 ps – 500 ps) a transient signal persists but its rate of decay does not show a *Q*-factor dependence, *i.e.* the decay curves have a constant gradient after the log-break in **a** and **b**. **c.** Decay dynamics for n = 3.5 mirror pairs microcavity as a function of the intensity of the exciting laser pulse. The dynamics appear independent of the laser fluence in the range applied. **d.** Decay dynamics of bare (~150 nm thick) R-BODIPY film as a function of laser fluence, revealing clear power dependence.

S4: Extraction of $\sigma(t)$

We can define σ as the distance between the centre of the signal and the point in the image plane (in any direction) where the signal strength is $e^{-1/2}$ of that at the centre. Mathematically this is expressed by

$$\sigma^2(\theta,t) = \left(x_{\frac{1}{2}}(\theta,t) - x_0\right)^2 + \left(y_{\frac{1}{2}}(\theta,t) - y_0\right)^2$$

where (x_0, y_0) and $(x_{\frac{1}{2}}(\theta,t), y_{\frac{1}{2}}(\theta,t))$ are the coordinates of the signal centre and the points where the signal strength at time t drops to $e^{-1/2}$ of that at the centre at angle θ relative to the positive x-axis, respectively. We will refer to $(x_{\frac{1}{2}}(\theta,t), y_{\frac{1}{2}}(\theta,t))$ as the 'sigma points' from now on. Extracting σ from the experimental data allows monitoring of the spatial movement of population as a function of time.

To extract σ from our experimental data we must first extract the centre of our signal. It is tempting to use the pixel with the strongest signal in each frame as the centre, as one might expect its position to be constant over time. However, since one pixel on the EMCCD camera corresponds to 55.5 × 55.5 nm^2 in real space and the pump spot has a FWHM of ~270 nm, the central four pixels often receive similar numbers of photons. In practice, the position of the maximum signal pixel fluctuates between 1-2 pixels in either direction. This is undesirable as physically the centre of the signal is defined as the centre of the pump pulse, which has a well-defined position that stays constant throughout the measurement window. To overcome this we utilise a number of pixels around the maximum to estimate the centre through a weighted average. It works almost identically to calculating the centre of mass (CoM) of N discrete masses on a plane, which can be expressed algebraically using the following equations:

$$X_{CoM} = \frac{1}{M}\sum_{i=1} m_i x_i$$
$$Y_{CoM} = \frac{1}{M}\sum_{i=1} m_i y_i$$

where $(X,Y)_{CoM}$ is the coordinates of the centre of mass, M is the total mass, m_i and (x_i, y_i) are the mass and coordinates of the i-th individual mass respectively. To calculate the centre of our signal, we substitute the Δ*T/T* data points for the individual masses in a 10 × 10 square window surrounding the maximum signal pixel (*i.e.* $i_{max} = 100$). We can then average the CoM over several frames and calculate its uncertainty in a similar fashion.

After we have estimated the signal centre, we need to extract the sigma points (i.e. coordinates where the signal strength is $e^{-1/2}$ of that at the centre), at any angle and time with sub-pixel resolution. This is achieved by mapping the data onto a fine polar grid centred at $(X,Y)_{CoM}$, followed by selecting in each direction the point which has an amplitude closest to $e^{-1/2}$ of that at the centre. $\sigma^2(\theta,t)$ can then be calculated.

Finally, we need to define time zero (*i.e.* the moment when the pump pulse arrives at the sample), for which three common definitions exist: Here, t_0 is placed when the Δ*T/T* signal is at the maximum. This is where σ is found to be closest to the diffraction-limited pump spot size ($2\sigma \approx 1.7 \times FWHM \approx 1.7 \times \frac{\lambda}{1.1NA}$) and avoids any potential artefacts (coherent artefact, pump scatter, *etc*) from the arrival of the pump pulse.

S5: Pump-probe spot size at t_0

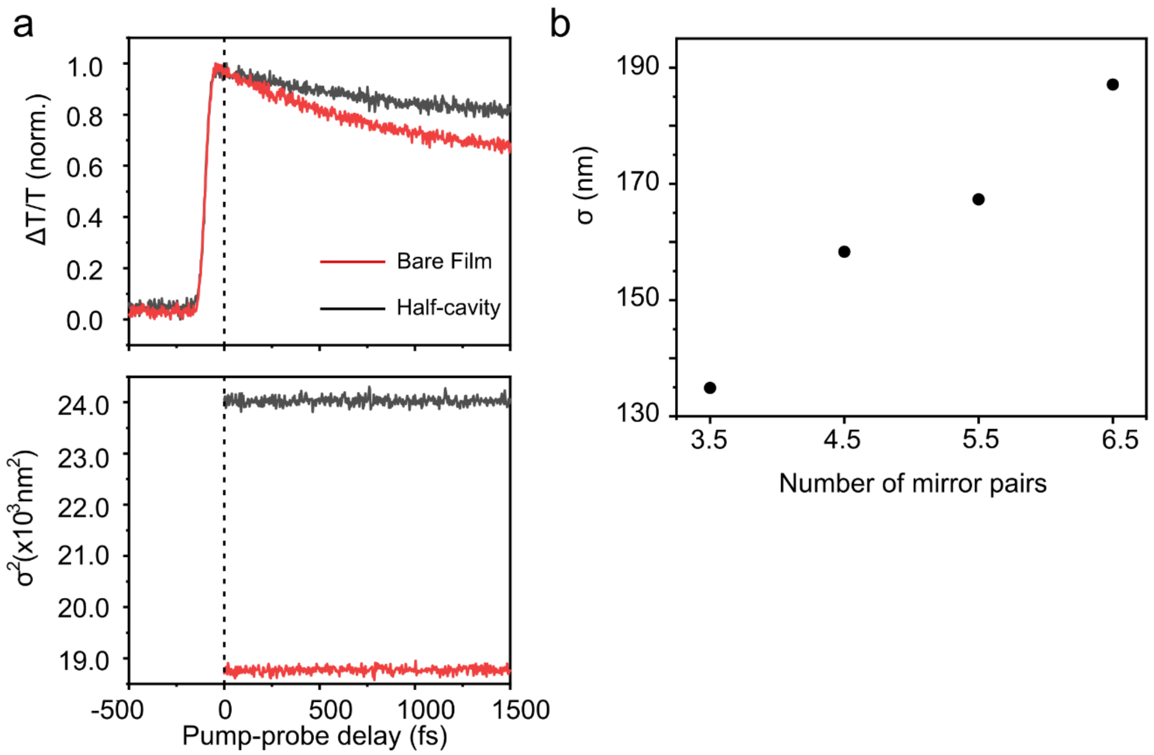

Figure S5. fs-TAM of bare BODIPY-R films and half-cavities. a. fs-TAM decay kinetics from bare BODIPY-R films and half-cavity (BODIPY-R film on a 3.5-pair SiO_2/Nb_2O_5 stack). The lifetime is taken at the GSB of the signal ~640 nm. The MSD traces for both remain flat over the first 1.5 ps confirming no spatial transport occurs in this regime in the absence of polaritons. **b.** Spot size at t_0 (σ) increases with the number mirror pairs due to refractive index mismatch between microscope oil and cavity which increases with n (see below). This systematic artefact is directly accounted for in our analysis and does not affect the conclusions drawn.

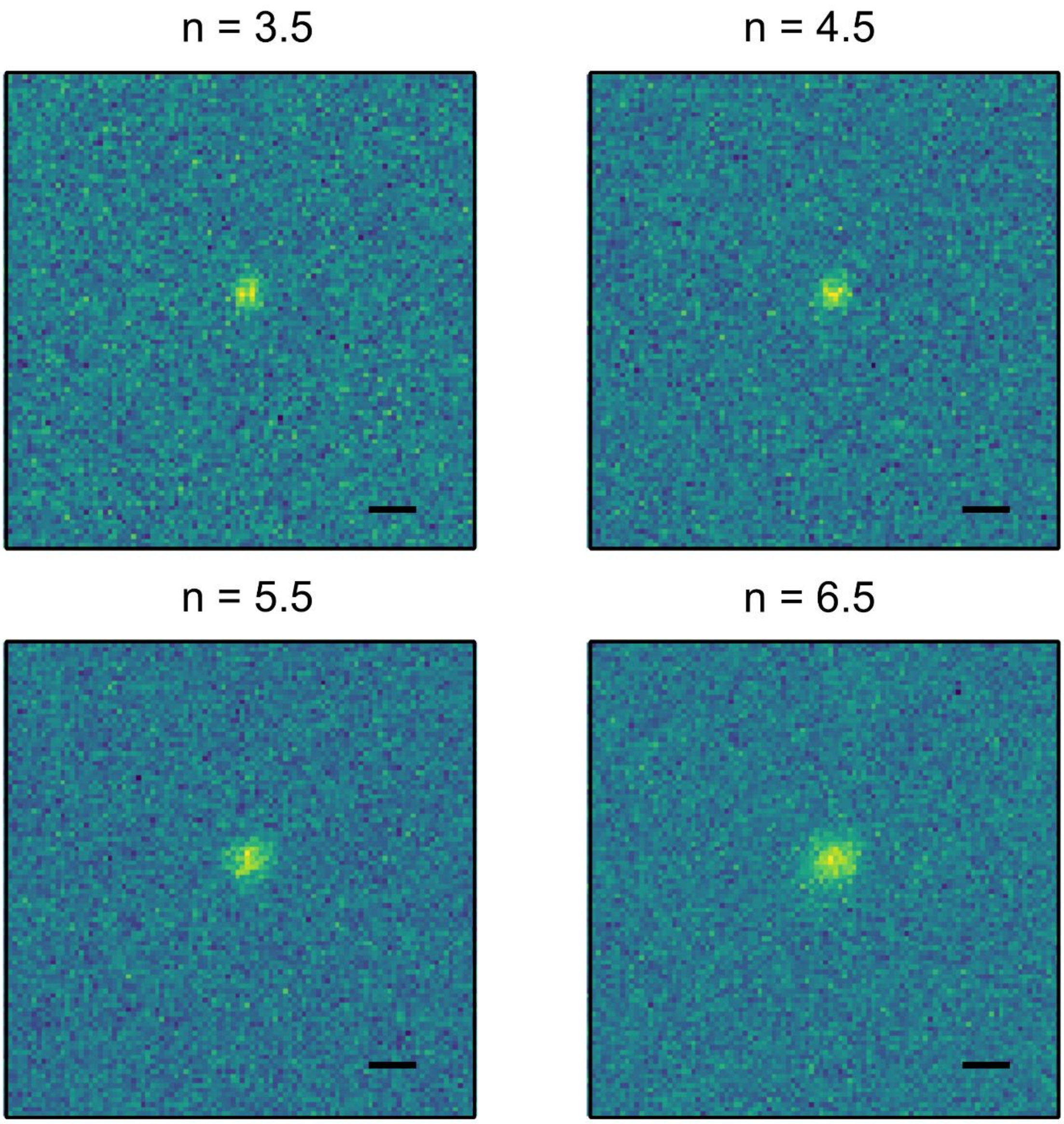

Figure S6. Variation of excitation spot size with mirror thickness. TAM images of the tightest observable reflected pump spot from microcavities of varying numbers of mirror pairs n. The size of the reflected spot increases with n, due to the refractive index effects discussed above and in the main text. Scale bar in each panel is 500 nm.

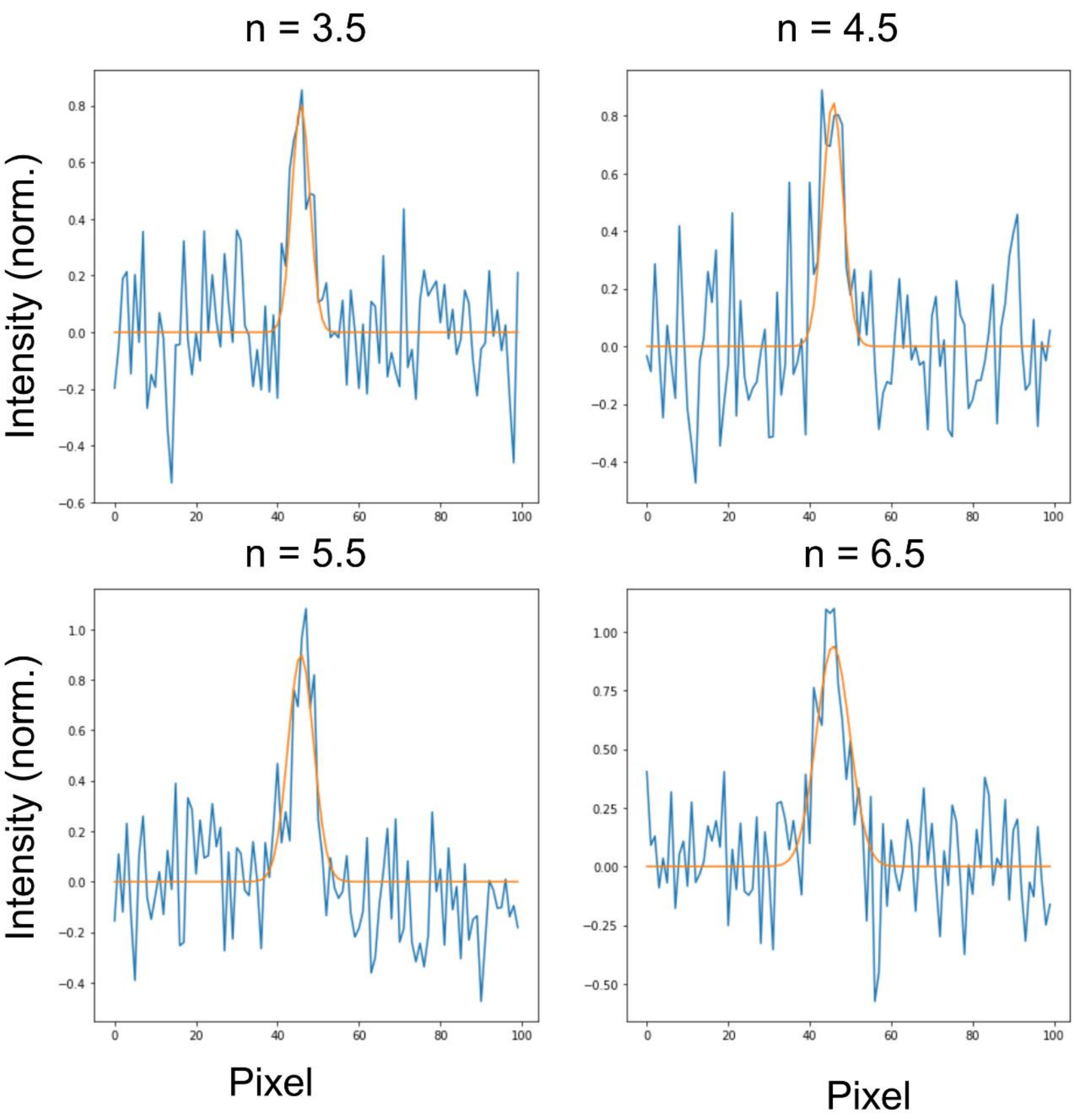

Figure S7. Independent characterization of excitation spot size. Photoluminesence intensity profile obtained on scanning pump pulse over a fluorescent bead (200 nm) on half-cavities of varying number of mirror pairs n. Blue shows raw data and orange a Gaussian fit. By deconvolving the spatial profile from the known diameter of the bead, the size of the pump spot can be obtained. The size of the imaged pump spot (varying between 130 ± 10 nm and 190 ± 10 nm in σ (see above)), though nominally fixed by our objective N.A. to a diffraction limit of ~135 nm in σ, varies precisely as described above, confirming the validity of our assignment of the polariton population size at t_0 in each cavity.

S6: Modelling coherent polariton propagation

At long times an initial Gaussian population will follow diffusive dynamics due to multiple scattering events effectively randomising the velocities of the carriers and giving the overall profile the dynamics of a Markovian random walk. It can be shown in this $t >> \tau$ limit that the characteristic diffusion constant in a 1D system appears as,

$$\sigma^2 = \sigma_0^2 + 2Dt$$

It is desirable to have a similar $\sigma(v,t)$ for the coherent transport regime in order to reliably extract the coherent group velocities v from the $\sigma(t)$. This is derived below.

We assume our microcavity has the simplest possible 1D band structure - a two band model for the ground state and the polariton branch. We further assume that we are exciting with a delta function like excitation. We assume the excitonic bands are sufficiently flat that the overall dispersion obeys inversion symmetry and the two k states we are exciting are at exactly +k' and -k'. As we are exciting states of a determinate k, they have a well-defined group velocity v_g.

Our initial excitation is a Gaussian with a known σ_0 with half the excitation having a velocity $+v_g$ and the other half a velocity $-v_g$. Hence we can decompose our initial excitation's time evolution as,

$$n(x,t) = \frac{1}{2\sigma\sqrt{2\pi}}\left(\exp\left[-\frac{(x-v_g t)^2}{2\sigma_0^2}\right] + \exp\left[-\frac{(x+v_g t)^2}{2\sigma_0^2}\right]\right)$$

Examining the above equation it is conceivable that in the limit $v_g t/\sigma_0 << 1$ the profile still appears Gaussian and we still have an effective σ. As coherent transport velocities are typically $< 10^6$ m s^{-1} = 1 nm fs^{-1}, $\sigma_0 \approx 100$ nm for a diffraction limited spot and the times range in which this physics is relevant is ≤ 50 fs at room temperature. For these parameters $v_g t/\sigma_0 < 1$ is well justified. Experimentally it is also well known that in this regime our profiles are Gaussians and we can find $\sigma(t)$. This expression therefore requires a Taylor expansion in $\frac{v_g t}{\sigma_0}$.

$$\begin{aligned}
n(x,t) &= A\left[\exp\left[-\frac{(x-v_g t)^2}{2\sigma_0^2}\right] + \exp\left[-\frac{(x+v_g t)^2}{2\sigma_0^2}\right]\right] \\
&= A\sum_{i=0}^{\infty}\left(-\frac{1}{2}\right)^i \frac{1}{i!}\left[\left(\frac{x-vt}{\sigma_0}\right)^{2i} + \left(\frac{x+vt}{\sigma_0}\right)^{2i}\right] \\
&= A\sum_{i=0}^{\infty}\left(-\frac{1}{2}\right)^i \frac{1}{i!}\left[\left(\frac{x^2-2xvt+v^2t^2}{\sigma_0^2}\right)^{i} + \left(\frac{x^2+2xvt+v^2t^2}{\sigma_0^2}\right)^{i}\right] \\
&= A\sum_{i=0}^{\infty}\left(-\frac{1}{2}\right)^i \frac{1}{i!}\left[\sum_{a+b+c=i} {}^iC_{a,b,c}\left(\frac{x^2}{\sigma_0^2}\right)^a\left(\frac{-2xvt}{\sigma_0^2}\right)^b\left(\frac{v^2t^2}{\sigma_0^2}\right)^c + \sum_{a+b+c=i} {}^iC_{a,b,c}\left(\frac{x^2}{\sigma_0^2}\right)^a\left(\frac{2xvt}{\sigma_0^2}\right)^b\left(\frac{v^2t^2}{\sigma_0^2}\right)^c\right] \\
&= A\sum_{i=0}^{\infty}\left(-\frac{1}{2}\right)^i \frac{1}{i!}\left[\sum_{a+b+c=i} {}^iC_{a,b,c}\left(\frac{x^2}{\sigma_0^2}\right)^a\left(\frac{v^2t^2}{\sigma_0^2}\right)^c\left[\left(-\frac{2xvt}{\sigma_0^2}\right)^b + \left(\frac{2xvt}{\sigma_0^2}\right)^b\right]\right]
\end{aligned}$$

This expansion's terms are zero for odd b, hence setting $b = 2b$ we collect the relevant terms

$$
\begin{aligned}
n(x,t) &= 2A\sum_{i=0}^{\infty}\left(-\frac{1}{2}\right)^{i}\frac{1}{i!}\left[\sum_{a+2b+c=i} {}^{i}C_{a,2b,c}\left(\frac{x^2}{\sigma_0^2}\right)^{a}\left(\frac{2xvt}{\sigma_0^2}\right)^{2b}\left(\frac{v^2t^2}{\sigma_0^2}\right)^{c}\right] \\
&= 2A\sum_{i=0}^{\infty}\left(-\frac{1}{2}\right)^{i}\frac{1}{i!}\left[\sum_{a+2b+c=i}\frac{i!}{a!\,(2b)!\,c!}\left(\frac{x^2}{\sigma_0^2}\right)^{a}\left(\frac{4x^2v^2t^2}{\sigma_0^4}\right)^{b}\left(\frac{v^2t^2}{\sigma_0^2}\right)^{c}\right] \\
&= 2A\sum_{i=0}^{\infty}\left(-\frac{1}{2}\right)^{i}\frac{1}{i!}\left[\sum_{a+2b+c=i}\frac{i!}{a!\,(2b)!\,c!}\left(\frac{x^2}{\sigma_0^2}\right)^{a}\left(\frac{4x^2}{\sigma_0^2}\right)^{b}\left(\frac{vt}{\sigma_0}\right)^{2(b+c)}\right]
\end{aligned}
$$

Knowing that $\frac{vt}{\sigma_0}$ is small we expand to the lowest relevant order. It is also important to note here that $a,2b,c \geq 0$ as required by the multinomial theorem. The zeroth order of $\left(\frac{vt}{\sigma_0}\right)^{2(b+c)}$ sets $b + c = 0$. As $b,c \geq 0$ naturally the only possible solutions are $b = 0$, $c = 0$ which sets $a = i$ for $i \geq 0$. The next order of $\left(\frac{vt}{\sigma_0}\right)^{2(b+c)}$ is second order in $\frac{vt}{\sigma_0}$ sets $b + c = 1$. As $b,c \geq 0$ the only possible solutions are $b = 1, c = 0$ and $b = 0, c = 1$. Using the constraint $a + 2b + c = i$ gives us tuples $(a,2b,c) = (i - 2,2,0)$ for $i \geq 2$ and $(a,2b,c) = (i - 1,0,1)$ for $i \geq 1$. Collecting these terms and discarding terms $O\left(\left(\frac{vt}{\sigma_0}\right)^{4}\right)$ we have

$$
\begin{aligned}
n(x,t) &= 2A\sum_{i=0}^{\infty}\left(-\frac{1}{2}\right)^{i}\frac{1}{i!}\left[\sum_{a+2b+c=i}\frac{i!}{a!\,(2b)!\,c!}\left(\frac{x^2}{\sigma_0^2}\right)^{a}\left(\frac{4x^2}{\sigma_0^2}\right)^{b}\left(\frac{vt}{\sigma_0}\right)^{2(b+c)}\right] \\
&\approx \underbrace{2A\sum_{i=0}^{\infty}\left(-\frac{1}{2}\right)^{i}\frac{1}{i!}\left(\frac{x^2}{\sigma_0^2}\right)^{i}}_{(a,2b,c)=(i,0,0)} + \underbrace{2A\sum_{i=1}^{\infty}\left(-\frac{1}{2}\right)^{i}\frac{1}{i!}\left[\frac{i!}{(i-1)!}\left(\frac{x^2}{\sigma_0^2}\right)^{i-1}\left(\frac{vt}{\sigma_0}\right)^{2}\right]}_{(a,2b,c)=(i-1,0,1)} + \\
&\qquad \underbrace{2\,A\sum_{i=2}^{\infty}\left(-\frac{1}{2}\right)^{i}\frac{1}{i!}\left[\frac{i!}{(i-2)!\,2!}\left(\frac{x^2}{\sigma_0^2}\right)^{i-2}\left(\frac{4x^2}{\sigma_0^2}\right)\left(\frac{vt}{\sigma_0}\right)^{2}\right]}_{(a,2b,c)=(i-2,2,0)} \\
&= 2A\exp\left[-\frac{x^2}{2\sigma_0^2}\right] - A\left(\frac{vt}{\sigma_0}\right)^{2}\underbrace{\sum_{i=1}^{\infty}\left(-\frac{1}{2}\right)^{i-1}\frac{1}{(i-1)!}\left(\frac{x^2}{\sigma_0^2}\right)^{i-1}} +
\end{aligned}
$$

set i-1 = i

$$
\left(\frac{x^2}{\sigma_0^2}\right)A\left(\frac{vt}{\sigma_0}\right)^{2}\underbrace{\sum_{i=2}^{\infty}\left(-\frac{1}{2}\right)^{i-2}\frac{1}{(i-2)!}\left(\frac{x^2}{\sigma_0^2}\right)^{i-2}}
$$

set i-2 = i

$$
= 2A\exp\left[-\frac{x^2}{2\sigma_0^2}\right] - A\left(\frac{vt}{\sigma_0}\right)^{2}\exp\left[-\frac{x^2}{2\sigma_0^2}\right] + \left(\frac{x^2}{\sigma_0^2}\right)A\left(\frac{vt}{\sigma_0}\right)^{2}\exp\left[-\frac{x^2}{2\sigma_0^2}\right]
$$

Defining a Gaussian that is not normalised as,

$$
\mathcal{G}(A,\mu,\sigma) = A\exp\left[-\frac{(x-\mu)^2}{2\sigma_0^2}\right]
$$

we can write,

$$
n(x,t) = \underbrace{\mathcal{G}(2A,0,\sigma_0)}_{A} - \underbrace{\mathcal{G}\left(A\left(\frac{vt}{\sigma_0}\right)^{2},0,\sigma_0\right)}_{B} + \underbrace{\left(\frac{x^2}{\sigma_0^2}\right)\mathcal{G}\left(A\left(\frac{vt}{\sigma_0}\right)^{2},0,\sigma_0\right)}_{C}
$$

A is simply the unperturbed Gaussian sitting at $\mu = 0$ which defines the overall TAM profile as Gaussian even in the coherent transport regime. It is expected that we would have such a term as we are expanding for small

changes around this profile. B is an inverted Gaussian that is responsible for reducing the maximum value of our Gaussian by a small amount. This results in a 'dip' in the centre of the profile that we usually cannot resolve. C is what is of interest to us as it represents a Gaussian that is being 'stretched out' by a term in x^2. One naturally expects C to capture to lowest order in vt/σ_0 the changes in σ of the overall profile.

We begin to try and find an effective σ for this distribution by starting with the ansatz,

$$n_{ansatz}(x,t) = A' \exp\left[-\frac{(x-\mu)^2}{2\sigma(t)^2}\right]$$

We set $\mu = 0$ for simplicity and write $\sigma(t) = \sigma_0 + f(v,t)$, where $f(v,t)$ will capture the effective small changes in σ due to coherent transport. We can naturally also make the approximation $f(v,t)/\sigma_0 << 1$ that results from the approximation $v_g t/\sigma_0 < 1$.

$$\begin{aligned}
n_{ansatz}(x,t) &= A' \exp\left[-\frac{x^2}{2\sigma(t)^2}\right] \\
&= A' \sum_{i=0}^{\infty} \left(-\frac{1}{2}\right)^i \frac{1}{i!} \left(\frac{x^2}{(\sigma_0 + f(v,t))^2}\right)^i \\
&= A' \sum_{i=0}^{\infty} \left(-\frac{1}{2}\right)^i \frac{1}{i!} (x^{2i})(\sigma_0 + f(v,t))^{-2i} \\
&= A' \sum_{i=0}^{\infty} \left(-\frac{1}{2}\right)^i \frac{1}{i!} \left[\frac{x^2}{\sigma_0^2}\right]^i \left[1 + \frac{f(v,t)}{\sigma_0}\right]^{-2i} \\
&= A' \sum_{i=0}^{\infty} \left(-\frac{1}{2}\right)^i \frac{1}{i!} \left[\frac{x^2}{\sigma_0^2}\right]^i \left[1 - 2i\frac{f(v,t)}{\sigma_0} + O\left(\frac{f(v,t)^2}{\sigma_0^2}\right)\right] \\
&= \mathcal{G}(A', 0, \sigma_0) - \underbrace{2A' \sum_{i=0}^{\infty} \left(-\frac{1}{2}\right)^i \frac{1}{i!} \left[\frac{x^2}{\sigma_0^2}\right]^i \left(i\frac{f(v,t)}{\sigma_0}\right)}
\end{aligned}$$

I = 0 term is 0

$$\begin{aligned}
&= \mathcal{G}(A', 0, \sigma_0) - 2A' \sum_{i=1}^{\infty} \left(-\frac{1}{2}\right)^i \frac{1}{(i-1)!} \left[\frac{x^2}{\sigma_0^2}\right]^i \left(\frac{f(v,t)}{\sigma_0}\right) \\
&= \mathcal{G}(A', 0, \sigma_0) + \left[\frac{x^2}{\sigma_0^2}\right] A' \left(\frac{f(v,t)}{\sigma_0}\right) \underbrace{\sum_{i=1}^{\infty} \left(-\frac{1}{2}\right)^{i-1} \frac{1}{(i-1)!} \left[\frac{x^2}{\sigma_0^2}\right]^{i-1}}
\end{aligned}$$

set i-1 = i

$$= \underbrace{\mathcal{G}(A', 0, \sigma_0)}_{D} + \underbrace{\left(\frac{x^2}{\sigma_0^2}\right) \mathcal{G}\left(A'\left(\frac{f(v,t)}{\sigma_0}\right), 0, \sigma_0\right)}_{E}$$

Comparing the expansion of the n_{ansatz} with n, it is clear that the term A and D are the same Gaussian by setting $A^0 = 2A$ as expected. It is not surprising that we were not able to capture the term B with the Ansatz as it is a single Gaussian and cannot have any 'dips' in the middle caused by the term B - we also note that this term is a Gaussian of with a maximum amplitude $\left(\frac{vt}{\sigma_0}\right)^2$ lower than the main Gaussian. Finally and most importantly, comparing terms C and E we see clearly that they both are of the form $\left[\frac{x^2}{\sigma_0^2}\right]\mathcal{G}$ differing by a factor of 2. Absorbing the factor of 2 into the gaussian and setting these terms as equal we get,

$$A'\left(\frac{f(v,t)}{\sigma_0}\right) = A\left(\frac{vt}{\sigma_0}\right)^2$$

$$2A\left(\frac{f(v,t)}{\sigma_0}\right) = A\left(\frac{vt}{\sigma_0}\right)^2 \implies f(v,t) = \frac{v^2t^2}{\sigma_0}$$

$$\implies \boxed{\sigma(v,t) = \sigma_0 + \frac{v^2t^2}{2\sigma_0}}$$

We can add in scattering by hand as an exponential pre-factor the velocity term and write,

$$\sigma(v,t) = \sigma_0 + \exp(-t/\tau)\left(\frac{v^2t^2}{2\sigma_0}\right)$$

References

[1] S. A. Furman, A. V. Tikhonravov, in *Basics Opt. Multilayer Syst.*, Atlantica Séguier Frontières, Paris, **1992**, pp. 1–102.

[2] D. G. Lidzey, D. M. Coles, in *Org. Hybrid Photonic Cryst.*, Springer International Publishing, Cham, **2015**, pp. 243–273.

[3] T. Yagafarov, D. Sannikov, A. Zasedatelev, K. Georgiou, A. Baranikov, O. Kyriienko, I. Shelykh, L. Gai, Z. Shen, D. Lidzey, P. Lagoudakis, *Commun. Phys.* **2020**, *3*, 1.

[4] S. Renken, R. Pandya, K. Georgiou, R. Jayaprakash, L. Gai, Z. Shen, D. G. Lidzey, A. Rao, A. J. Musser, *J. Chem. Phys.* **2021**, *155*, 154701.
